# Dexamethasone Treatment Limits Efficacy of Radiation, but Does Not Interfere With Glioma Cell Death Induced by Tumor Treating Fields

**DOI:** 10.3389/fonc.2021.715031

**Published:** 2021-07-30

**Authors:** Benedikt Linder, Abigail Schiesl, Martin Voss, Franz Rödel, Stephanie Hehlgans, Ömer Güllülü, Volker Seifert, Donat Kögel, Christian Senft, Daniel Dubinski

**Affiliations:** ^1^Experimental Neurosurgery, Neuroscience Center, Goethe University Hospital, Frankfurt, Germany; ^2^Dr. Senckenberg Institute of Neurooncology, Goethe University Hospital, Frankfurt, Germany; ^3^Department of Radiotherapy and Oncology, Goethe University Hospital Frankfurt, Frankfurt, Germany; ^4^Department of Neurosurgery, Goethe University Hospital, Frankfurt, Germany

**Keywords:** brain cancer, TTFields, corticosteroids, dexamethasone, glioma, survival, translational investigation

## Abstract

**Purpose:**

Dexamethasone (Dex) is the most common corticosteroid to treat edema in glioblastoma (GBM) patients. Recent studies identified the addition of Dex to radiation therapy (RT) to be associated with poor survival. Independently, Tumor Treating Fields (TTFields) provides a novel anti-cancer modality for patients with primary and recurrent GBM. Whether Dex influences the efficacy of TTFields, however, remains elusive.

**Methods:**

Human GBM cell lines MZ54 and U251 were treated with RT or TTFields in combination with Dex and the effects on cell counts and cell death were determined *via* flow cytometry. We further performed a retrospective analysis of GBM patients with TTFields treatment +/- concomitant Dex and analysed its impact on progression-free (PFS) and overall survival (OS).

**Results:**

The addition of Dex significantly reduced the efficacy of RT in U251, but not in MZ54 cells. TTFields (200 kHz/250 kHz) induced massive cell death in both cell lines. Concomitant treatment of TTFields and Dex did not reduce the overall efficacy of TTFields. Further, in our retrospective clinical analysis, we found that the addition of Dex to TTFields therapy did not influence PFS nor OS.

**Conclusion:**

Our translational investigation indicates that the efficacy of TTFields therapy in patients with GBM and GBM cell lines is not affected by the addition of Dex.

## Introduction

### Dexamethasone Administration for Vasogenic Edema Management in Patients With Glioblastoma

Patients suffering from glioblastoma (GBM) usually are afflicted with perilesional edema that is caused by a tumor-induced disruption of the blood brain barrier (BBB) (1). Defective astrocytes lead to the impairment of endothelial tight junctions on the one hand and tumor-produced vascular endothelial growth factor (VEGF) that increases vessel permeability on the other hand resulting in the diffusion of fluid into the extracellular brain parenchyma with a consequent increase of intracranial pressure (ICP) (2). The resulting perilesional edema is the major contributor to patient’s neurologic deficits. Corticosteroids reduce the permeability of tumor vessels by upregulating tight junctions and inducing the transcription of several genes that are involved in stabilization of the BBB (e.g. occludin, NF-κB, VE-cadherin etc) (3). In the clinical practice, Dexamethasone (Dex) has become the corticoid of choice for brain tumor-associated cerebral edema due to its fast and effective alleviation of perilesional edemas, long half-life, low mineralocorticoid activity and the reduction of nausea. Despite its routine clinical use, the lack of prospective clinical studies impairs the implementation of a standard dosage protocol. Usually, the orally administered dosage ranges from daily 2 to 20 mg Dex (4).

### Unfavorable Clinical Effects of Concomitant Dex Administration in GBM

Given the fact that perilesional edemas are a major cause of mortality in GBM patients, their treatment is indispensable. However, long-term Dex ingestion also leads to numerous well characterized clinical side effects including insomnia, psychiatric alterations, tremor, hyperglycemia, muscle atrophy, cushingoid appearance, hypertension, gastrointestinal perforation and immunosuppression (1). Furthermore, recent studies identified concomitant Dex administration as a risk factor for an impaired progression-free (PFS) and overall survival (OS) of patients suffering from GBM. A retrospective clinical study of 73 GBM patients demonstrated that Dex administration concomitant to radiation therapy (RT) leads to a reduction of the OS from 22.6 to 12.7 months (4). Furthermore, a multicentre retrospective analysis of more than 2000 GBM patients identified Dex as an independent risk factor for poor outcome, even after adjusting for extent of resection, initial treatment, age and Karnofsky Performance Score (KPS) (5). Another study demonstrated that patients with Dex-induced leucocytosis (DIL) had decreased OS and PFS and showed a significant reduction of tumor-infiltrating leukocytes and lymphocytes (6).

### Experimental Effects of Dex Administration in Preclinical Studies

The molecular effects of Dex on GBM cells as described in previous studies are pleiotropic and partially conflicting, possibly related to context-dependent effects in different tumors/cell models and experimental setups. Accordingly, Dex was shown either to inhibit or to stimulate the proliferation of glioma cells *in vitro.* Previous data further suggest a time-dependent and dosage-dependent antiproliferative effect of Dex (7, 8). Moreover, Dex administration was associated with reduced glioma cell invasion, primarily caused by decreased transcription of metalloproteases (9). On the other hand, Dex decreased the efficacy of chemotherapy by counteracting an alkylating agents induced apoptosis in primary GBM cell lines (10). The addition of Dex to glioma stem cells led to increased proliferation and invasion (2). In addition, Dex treatment leads to a decreased hypoxia-sensitivity in primary glioma cell lines, presumably by downregulation of VEGF (11).

### TTFields in GBM Therapy

TTFields (Optune, Novocure LTD) is a new type of cancer treatment modality that has been shown to significantly improve outcome in GBM patients in combination with radio-chemotherapy and was approved for newly diagnosed and recurrent GBM (12). TTFields create alternating electric fields with varying frequencies between 50 to 400 kHz and its efficacy is dependent on the cell type, size and orientation (13). Two pairs of juxtapositioned transduced arrays placed on the patient’s skin, deliver a locoregional antiproliferative and cell-killing effect on mitotic glioma cells by interfering with the cell’s mitotic apparatus (disruption of the polymerization of highly dynamic microtubules and septin filaments). This electromechanical cell cycle intervention leads to abnormal chromosome segregation and consecutive cell death (14). However, recent studies explored further mechanisms of action including the inhibition of the DNA damage response (DDR) by altered expression of DNA repair genes in the BRCA1 pathway and impaired cellular migration and invasion (15). Furthermore, TTFields increased immunogenic cell death in combination with anti-PD1 therapy presumably by increasing the amount of CD45+ tumor infiltrating cells.

### Aim of This Study

To date, Dex remains the gold standard of edema treatment in the clinical setting due to its highly effective resolution of perilesional edema and fast improvement of patient’s neurologic deficits despite the unfavorable long-term consequences. Previous studies identified the addition of Dex to increased radio resistance and poor outcome in glioma therapy. Furthermore, TTFields therapy is a novel effective treatment modality that shows improved survival in GBM therapy and is now widely used in the clinical setting. Yet, the effects of concomitant Dex administration during TTFields therapy remain unknown. We thus conducted this translational study to analyse the effects of Dex on TTFields efficacy in patients with GBM and GBM cell lines.

## Materials and Methods

### Patients and Data Collection

For this study, an ethical approval was obtained from ethics committee of the University hospital Frankfurt am Main (Identification number: 20-676). As a non-interventional, retrospective single-center study no patient consent was necessary.

### Patient Cohort

In total, 26 GBM patients that were treated at the Department of Neurosurgery, University Hospital, Goethe University Frankfurt am Main and received TTFields treatment between November 2015 and September 2019 were retrospectively analysed. According the EF 14 trial the inclusion criteria was pathological GBM verification, age over 18 years, Karnofsky scale ≥ 70, received maximal debulking surgery and radiotherapy concomitant with Temozolomide (45-70Gy). Further inclusion criteria were the application of TTFields (Novocure, LTD). Patients in the Dexamethasone cohort were identified as presence of Dexamethasone medication at the beginning of TTFields treatment and the dosage ranged between 0.5 and 4mg/d. Patient characteristics that were extracted from the medical chart included sex, age, MGMT methylation status, date of starting and ending TTFields therapy, date of surgery, date of death or date of last contact and the date of tumor progression. Tumor progression was defined as the date of cranial MRI with progressive disease according to the RANO 18 criteria and/or the assessment of the local interdisciplinary neurooncological tumor board (16).

### Clinical Application of TTFields

Within the framework of this trial, TTFields were started after completion of radiochemotherapy. The alternating electric fields were delivered (≥ 18 hours/d) *via* 4 transducer arrays on the shaved scalp. Temozolomide was administered (150-200 mg/m2) for 5 days per 28-day cycle (6-12 cycles) (12).

### GBM Cell Lines and Culture

U251-MG (U251) and MZ-54 (17), two adherent human Glioblastoma wild type cell lines were used. Both cell lines were maintained in DMEM Glutamax Media (Sigma-Aldrich) supplemented with 10% heat-inactivated FCS (Invitrogen) and 1% Penicillin/Streptomycin (Invitrogen). For cultivation, cells were kept in an incubator at 37°C and a 5% CO2 atmosphere. We passaged the cells weekly at a ratio of 1:10 for MZ-54 or 1:20 for U251 using Trypsin (Sigma, Aldrich) as detachment solution. A 100mg/10ml Dex stock injection solution (Jenapharm) was added after media change in final half-maximal inhibitory concentrations (IC50) of 65 µM for MZ-54 cells and 165 µM for U251 cells. Dex was kept at 4°C in a light sealed Falcon.

### TTFields Application

TTFields were applied according to the protocol described by Porat et al. (17) with minor modifications to the experimental setup. 10.000 cells per dish were seeded on 24 mm² coverslips in 500µl DMEM medium placed at the bottom of the ceramic TTFields dishes. After overnight incubation at 37°C, the medium was removed and replaced with fresh 2 ml DMEM medium with or without Dex. The TTFields dishes were covered with Parafilm (Sigma-Aldrich) manually before starting TTFields treatment. Cells were then subjected to electric field treatment at 250 kHz for MZ-54 and 200 kHz for U251 and expected intensities between 1.48 V/cm – 1.41 V/cm for 24h, 48h and 72h using the Inovitro™ system (Novocure Haifa, Israel). The TTFields dishes were kept inside an incubator at 20°C - 21°C, since the Novocure device produces excessive heat (18). After harvesting and Annexin/PI staining, the effects of TTFields on cell death induction and cell count were analysed by a BD Accuri C6 (BD Biosciences, Franklin Lakes, New Jersey, USA) fluorescence-activated cell-sorting device (FACS).

### Frequency Scan

For the determination of the optimal frequency, TTFields were administered as described in Porat et al. (17), on MZ-54 cells at different frequencies ranging from 200 kHz, 250 kHz, 300 kHz to 350 kHz for a duration of 72 h. Cell death and cell count were then determined *via* flow cytometry. For U251 cells, we worked with 200 kHz as optimal frequency as used in previous studies (19).

### Cell Viability Assay

The IC50 concentration of Dex were determined for four different cell lines (U251 and MZ-54) using the MTT-Assay. For this purpose, cells were plated at 5.000 cells/well in 96-well plates and a day later subjected to 72 h Dex treatment at increasing concentrations: 0 µM, 0.2 µM, 1 µM, 5 µM, 10 µM, 50 µM, 100 µM, 250 µM, 500 µM, 1 mM. Cells were cultivated at 37°C. At time points 0 h, 24 h, 48 h, 72 h the cell confluence was measured using the Tecan reader. For the determination of cell viability, 20 µl of 5 mg/ml MTT-Tetrazolium salt (3-[4,5-Dimethylthiazol-2-yl]-2,5-diphenyltetrazolium bromide) (Sigma-Aldrich) was added to each well after treatment. After allowing cells to incubate for 3 h at 37°C, the media containing MTT was carefully removed and 100 µl isopropanol/HCl solution (1ml HCl in 24 ml Isopropanol) was added to each well with gently mixing for 20 min to dissolve the formazan crystals and fixate the cells. The photometrical absorption was measured using a Tecan Spark plate reader (Tecan) at a wavelength of 560 nm.

### IC50 Calculations

The IC50 value is the concentration of a drug in which cell viability is inhibited to 50% of the control. The IC50 was determined by nonlinear regression analysis in GraphPad Prism (Version 7, GraphPad Software) using the function “log (inhibitor) *vs.* response (three parameters) of the data derived from the MTT measurement after normalizing the data from solvent-treated cells to 100% using the “remove baseline” function.

### Flow Cytometry

After the treatment period the medium was removed, and wells were washed with PBS. Cells were next trypsinized and incubated for 10 min at 37°C. PBS was added to the cells to stop trypsin reaction, washed twice, and then transferred into FACS tubes. The FACS tubes were centrifuged for 3 minutes at 195 x g to form pellets. After discarding the supernatant, cells were stained with 0.8 µl Propidium Iodide (Sigma-Aldrich, 10 µg/ml) and 0.8 µl Annexin V-APC (BD Pharmingen #550475) in 50 µl FACS-Buffer, mixed and incubated in the dark for 10 min at room temperature. Flow cytometric determination of cell death was performed by counting of 10.000 cells on an Acurri C6 (Becton Dikinson).

### Irradiation Procedures

Cells were plated in 12 well-plates and then pre-treated with or without Dex for 24 h prior to radiation. Irradiation (IR) was performed using a linear accelerator with 6 MV photon energy, 100 cm focus to isocentre distance and a dose rate of 6 Gy/min (Elekta, Crawley, UK) at the Department of Radiation Therapy (University Hospital Frankfurt, Frankfurt, Germany). GBM cells were irradiated at room temperature with a dose of 10, 20, 30, 40 Gy. Afterwards cells were incubated with or without Dex for another 48 h and 72 h at 37°C. Control cells underwent the same experimental conditions.

### Statistical Analysis

Statistical analysis was done using GraphPad Prism 7 (GraphPad Software, La Jolla CA, USA). The minimum level of statistical significance was set at p ≤ 0.05. Significances were marked as follows: p ≤ 0.05: *, p ≤ 0.01: **, p ≤ 0.001: ***, p<0.0001: ****, n.s. not significant. Significances are depicted between control and treatments or as indicated. To estimate the survival rates, the Kaplan-Meier analysis was used. The differences between curves were assessed using the log-rank test. Progression-free survival (PFS) was defined as the time from diagnosis to first recurrence or death. Overall survival (OS) was defined as the time of first presentation to death. The applied statistical test is denoted in the respective figure legend.

## Results

**Figure 1:**
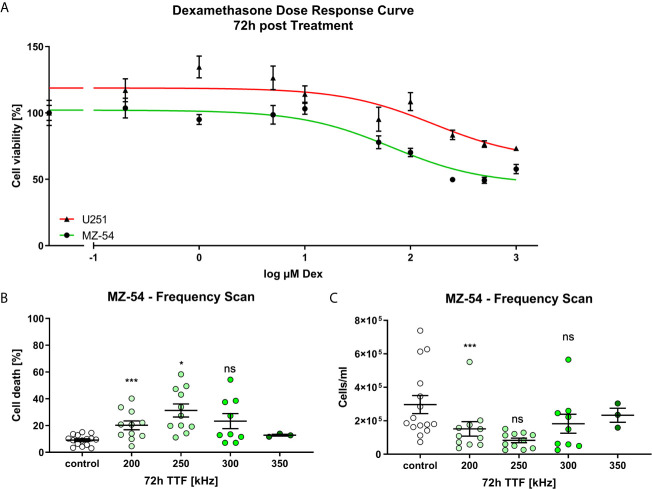
**(A)** Non-linear regression (log (inhibitor) *vs.* response (three parameters)) of MZ-54 (green) and U251 (red) 72h after treatment with increasing concentrations of Dex and determining cell viability using MTT Assay. The half-maximal inhibitory concentration was determined at 65 µM and 165 µM for MZ-54 and U251, respectively. **(B)** FACS-based measurement of cell death of MZ-54 cells 72h after Tumor Treating Field (TTFields) application at the depicted frequency. **(C)** FACS-based measurement of cell count derived from the same measurement as in **(B)**. The optimal frequency of 250 kHz was selected for MZ-54 cells. ns, not significant; *p < 0.05; **p < 0.01; ***p < 0.001; One-Way ANOVA with Dunnett’s multiple comparison test (GraphPad Prism 7).

In order to test our hypothesis whether Dex affects the efficacy of TTFields-treatment we first determined the response of the cells towards Dex. For this purpose, we treated the cells with increasing concentrations of Dex, ranging from 0.2 µM to 1000 µM and measured cell viability after 72 h using MTT assays (**Figure 1A**). Afterwards we determined the IC50 values using non-linear regression analyses and obtained an IC50 of 65 and 165 µM for MZ-54 and U251, respectively. This concentration reflects the frequently used clinical dosage of 4-16 mg/d. Next, we aimed to determine the optimal TTFields frequency for MZ-54 cells. Thus, we performed a frequency scan using 200, 250, 300 and 350 kHz of MZ-54 cells and measured cell death (**Figure 1B**) and cell count (**Figure 1C**). This approach revealed that the optimal frequency for MZ-54 cells is 250 kHz. For U251 cells we adopted the best frequency available from the literature at 200 kHz (20).

**Figure 2:**
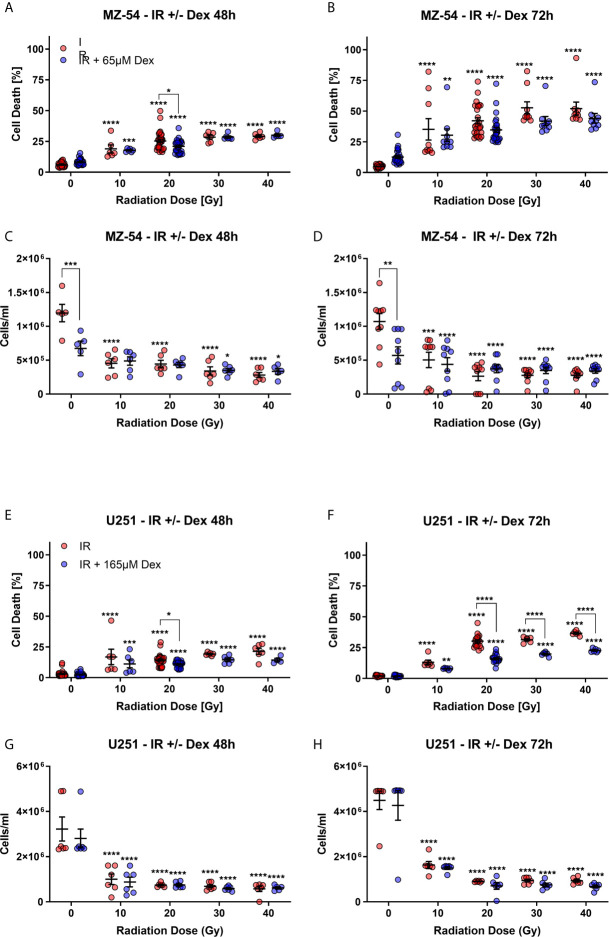
FACS-based measurement of **(A, B)** cell death and **(C, D)** cell count of MZ-54 GBM cells after 24h pre-treatment with Dex and irradiation (IR). The measurements were conducted **(A, C)** 48h and **(B, D)** 72h after IR-treatment. MZ-54 show dose-dependent increases in cell death and concomitant decreases in cell counts, with no discernible effect through the addition of Dex. FACS-based measurement of **(E, F)** cell death and **(G, H)** cell count after **(E, G)** 48h and **(F, H)** 72h of U251 GBM cells treated accordingly show similar IR-dose-dependent increases and decrease in cell death and cell count respectively. Note that U251 cells are protected from IR-induced cell death after additional Dex-treatment. *p < 0.05; **p < 0.01; ***p < 0.001; ****p < 0.0001; Two-Way ANOVA with Dunnett’s multiple comparison test (GraphPad Prism 7).

As outlined above, recently, it was shown that Dex can protect GBM cells from radiation-induced cell death *in vitro* (5). Therefore, we first wanted to test whether these effects using our cell models. For this purpose, we pre-treated MZ-54 and U251 GBM cells with Dex for 24 h before radiation treatment consisting of 10, 20, 30 and 40 Gy (**Figure 2**). After 48 h and 72 h after irradiation, cell death, and that after cell counts were determined *via* flow cytometry. These experiments showed that in MZ-54 cells (**Figures 2A, B**) increasing doses of radiation resulted in increased cell death, whereas after 72 h the amount of cell death was higher compared to 48 h. The addition of Dex had almost no statistically significant effect on cell death in MZ-54, except for 20 Gy after 48 h, where a moderate cell death rescue was observed. These observations are further corroborated by our analyses of the cell counts (**Figures 2C, D**). Here, we observed after both timepoints a Dex-induced decrease in cell number in non-irradiated control cells. In contrast, IR-treatment effectively and dose-dependently reduced the amount of cells significantly, but an additional Dex-treatment had no further inhibiting effect. U251 (**Figures 2E–H**) also showed a dose-dependent increase in cell death (**Figures 2E, F**) and reduction in cell number (**Figures 2G, H**) with stronger effects at the later time point. Dex alone had no discernible effect on either cell death or cell count in non-irradiated cells, whereas it could rescue cell death at 20 Gy IR after 48 h and even more pronounced at doses higher than 20 Gy after 72 h.

**Figure 3:**
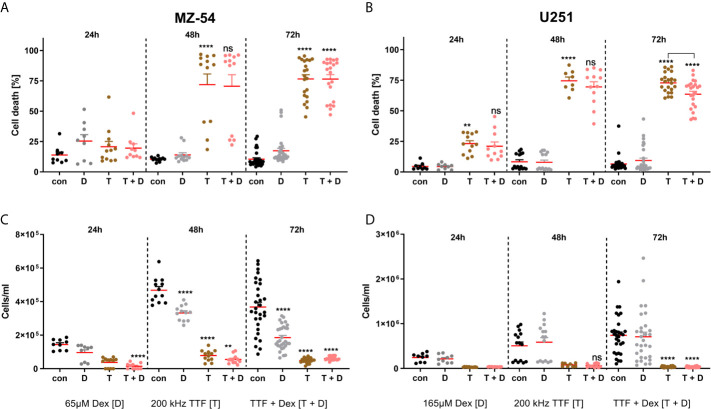
FACS-based measurement of **(A, B)** cell death and **(C, D)** cell count of **(A, C)** MZ-54 and **(B, D)** U251 GBM cells after treatment with the IC50 of Dex and Tumor Treating Field (TTFields) application at the optimal frequency for 24, 48 and 72h. Effective cell death induction can be appreciated after 48h using TTFields and TTFields + Dex in both GBM cell lines with a concomitant decrease in cell count. Note the MZ-54 also display reduced cell counts after 48h and 72h after Dex alone and U251 already display significantly induced cell death after 24h. *p < 0.05; **p < 0.01; ***p < 0.001; ****p < 0.0001; ns, non significant. One-Way ANOVA with Tukey’s multiple comparison test (GraphPad Prism 7). Con, control; D, dex; T, TTFields; T + D, TTFields + Dex.

Next, we wondered if Dex has a similar effect on cell death induction of TTFields treatment. For this purpose, we treated the cells with IC50 concentrations of Dex and simultaneously commenced TTFields treatment (**Figure 3**) for 24, 48 and 72 h and measured cell death and cell counts *via* flow cytometry. This analysis revealed that in treated MZ-54 cells (**Figure 3A**) no cell death occurred at 24 h, whereas after 48 and 72 h cell death was very pronounced. The addition of Dex had neither a discernible effect on TTFields efficacy nor on its own. For U251 (**Figure 3B**), we could determine a significant induction of cell death after 24 h of treatment, which was strongly increased after 48 h and 72 h. The concurrent addition of Dex had no effect on cell death induction after 24 h and 48 h, but resulted in a moderate, yet significant, prevention of cell death after 72 h. Conversely, we also analysed cell count from our FACS data (**Figure 3C**). This analysis revealed that after 24h of treatment a slight downward-trend using Dex alone and TTFields alone for MZ-54 can be observed, which culminates in a significant reduced cell number in the combined treatment. At later time points (48h and 72h), the growth-inhibitory effects of Dex and TTFields became even more apparent, whereas TTFields treatment was more effective than Dex. The combined treatment showed slightly less cell numbers after 48h; however, this difference did not reach statistical significance and after 72h no difference was visible. Similar results regarding TTFields and combination treatment were obtained in U251 (**Figure 3D**), which can both effectively reduce the cell number with the effect being most pronounced after 72h. In contrast, Dex single treatment had no effect on cell number after any time point, which is in line with the reduced sensitivity observed using the dose-response curve, that MZ-54 are more sensitive towards Dex. Based on these results we concluded that Dex does not interfere with TTFields treatment *in vitro*. We further concluded that TTFields treatment may exhibit cell death to a greater extent compared to IR-treatment, especially in IR-resistant cell models such as U251.

**Figure 4:**
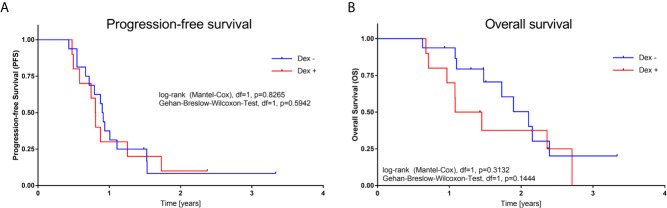
**(A)** Kaplan–Meier plots for progression-free survival **(A)**, and overall survival **(B)** stratified by Dex administration during TTFields treatment. P-values calculated from log-rank and Gehan-Breslow-Wilcoxon-test) (GraphPad Prism 7).

To crosscheck these findings in the clinical setting, we analysed the characteristics and clinical outcome of 26 patients that were treated for primary GBM in our University Hospital according to the EF-14 trial (12). During the TTFields therapy 10 patients received Dex and 16 patients had no concomitant Dex administration. The median Dex dosage was 2 mg (SD: 1.45). Male to female ratio was non-significant in our cohort (60% male in the Dex group *vs.* 75% in the Dex negative group). Median age was also statistically non-significant between the cohort (55 years in the Dex *vs.* 50 years in the Dex negative group). Furthermore, MGMT promotor methylation was observed in 50% of the patients in the Dex group *vs.* 44% in the Dex negative group. Finally, the median TTFields treatment time in days and the median day from operation to TTFields therapy was not-significant between the two cohort (**Table 1**). In addition, PFS was 9 months in the Dex cohort *vs.* 11 in patients without Dex treatment and thus not statistically significant. OS was 15 months in the Dex cohort *vs.* 18 months in patients without Dex again not reaching a level of significance (**Figure 4** and **Table 1**).

**Table 1:** Baseline characteristics of GBM cohort stratified by Dex administration during TTFields treatment.

Numbers	Dex =10	no Dex =16	*p*-value
**Sex**			
Male (n)	6 (60%)	12 (75%)	n.s.
Female (n)	4 (40%)	4 (25%)	n.s.
**Median age**			
Years	55 (23-75)	50 (27-68)	n.s.
**MGMT status**			
Methylated	5 (50%)	7 (44%)	n.s.
Unmethylated	5 (50%)	9 (56%)	n.s.
**IDH-1 status**			
Wildtype	9 (90%)	16 (100%)	n.s.
Mutated	1 (10%)	0 (0%)	n.s.
**P 53**			
Wildtype	6 (60%)	6 (38%)	n.s.
Mutated	4 (40%)	10 (62%)	n.s.
**Survival**			
Progression-free survival in months (range)	9 (5-28)	11 (5-39)	n.s.
Overall survival in months (range)	15 (8-32)	18 (7-39)	n.s.
**Median TTFields application in days**	177 (21-260)	92 (59-409)	n.s.
**Median Days from operation to TTFields**	163.5 (46.9)	175.5 (64.3)	n.s.

ns, non significant.

## Discussion

We aimed to determine whether concomitant Dex administration affects TTFields efficacy. We choose a translational approach to answer this question and found that Dex administration during TTFields application has no negative effect on the antitumor capacity *in vitro* and in a retrospective clinical evaluation. On the other hand, our *in vitro* results confirm the accumulating evidence against the usage of Dex during RT.

The addition of Dex during RT resulted a significantly increased radio-resistance in U251 GBM cells, whereas for MZ54 cells only a small tendency after 72h of treatment is apparent. These findings underscore the context-dependency of DEX effects that was also observed in other studies (5). This is in accordance with a clinical observation of Shields et al., who showed that Dex usage during RT was correlated with reduced OS and PFS (4). Additionally, Pitter et al., described Dex-induced anti-proliferative effects that may confer protection from radiotherapy-induced genotoxic stress, by inducing cell cycle arrest (5). In line with the mentioned research, our analysis confirm that Dex pre-treatment leads to a significant RT-induced cell death resistance in both GBM cell lines. Interestingly, this observed resistance does not occur when analysing the cell count, although an RT-dependent reduction in cell count can be observed. RT, first induces cell cycle arrest, which can ultimately lead to cell death if RT-induced damages cannot be restored and the cells proceed to cell cycle. Thus, we reason that the combined cell cycle arrest by Dex and RT more potently prevents the cells from escaping this cell cycle arrest, and therefore protects them from cell death.

On the other hand, *in vitro* TTFields application (200 kHz/250 kHz) for 72 h induced massive cell death in U251 and MZ54 cell lines. The frequency and efficacy of *in vitro* TTFields application is in line with the literature (17), while the higher frequency of MZ54 cells likely is due to their increased size compared to U251 cells.

In the clinical setting however, Dex weaning in symptomatic patients is problematic and administration is often maintained thorough adjuvant therapy. Therefore, the main question was whether concomitant Dex administration reduces TTFields efficacy analogous to RT in GBM. Adjusted for the optimal frequency and Dex concentration, the addition of Dex to TTFields showed no significant impact on cell death in MZ-54 and U251 cells. Complimentary, the retrospective analysis of GBM patients showed no significant impact on PFS and OS. Our study revealed no contraindication of Dex usage in GBM patients during TTFields application. However, these results should be evaluated in lager prospective clinical trials.

To answer the impending question why TTFields efficacy is not alternated by Dex is challenging. Wong et al. described their retrospective analysis of phase III registration trial comparing TTFields *vs* chemotherapy in recurrent GBM patients. Their unsupervised mathematical algorithm showed that a Dex dose higher than 4.1 mg per day was associated with reduced OS in the TTFields-treated cohort. Peripheral blood lymphocyte counts were independent of Dex application but positively correlated with patient’s outcome. The group therefore concludes that dexamethasone exerted a profound interference on the therapeutic effects of TTFields therapy (21). The median Dex dosage in our cohort was 2 mg and accordingly under the proposed cut-off. Collectively these data suggest that there could be a therapeutic window for concomitant DEX treatment without major effects on TTFields efficacy that can be used for the benefit of the GBM patients. We did not analyse peripheral blood lymphocytes which makes it difficult to oppose our studies. However, as TTFields are a local tumor therapy and its systemic effects remain elusive, we advocate the point of local antitumoral TTFields effect unaffected by Dex. The TTFields induced disruption of the mitotic chromosomes spatial order which results in asymmetric chromosome segregation and aneuploidy is supposedly not counteracted by systemic Dex administration.

Nevertheless, several studies identified high Dex dosage as prognostically unfavourable in GBM. We therefore advocate consequent Dex weaning where possible but our data indicates that concomitant application during TTFields therapy is not associated with poor efficacy and outcome.

Our study has several strengths and weaknesses. First, we analysed two cell lines, which cannot exclude different results in other cultivated GBM cells. As such, future research should include further cell lines including primary ones. As a strength, our investigation is the first study to answer the question of Dex effects by a translational approach and both *in vitro* and retrospective clinical findings resulted in coherent results. The obvious limitation of the clinical finding is the single centre character, the small sample size and the retrospective design. As this part is of observational character, confounding, selection bias, reverse causation and uncontrolled statistical error risk cannot be excluded. However, further prospective randomized trials with large cohorts are necessary to confirm our findings.

## Conclusion

This study provides the first evidence that concomitant Dex administration is not associated with reduced TTFields efficacy nor affects patient’s outcome in GBM therapy.

## Data Availability Statement

The raw data supporting the conclusions of this article will be made available by the authors, without undue reservation.

## Author Contributions

BL and AS collected the data. DD and BL wrote the first draft. DK, CS, and DD supervised the manuscript. All authors supplied additional information, edited the manuscript, and contributed to critical review and revision of the manuscript. All authors contributed to the article and approved the submitted version.

## Conflict of Interest

The authors declare that the research was conducted in the absence of any commercial or financial relationships that could be construed as a potential conflict of interest.

## Publisher’s Note

All claims expressed in this article are solely those of the authors and do not necessarily represent those of their affiliated organizations, or those of the publisher, the editors and the reviewers. Any product that may be evaluated in this article, or claim that may be made by its manufacturer, is not guaranteed or endorsed by the publisher.
